# Role of Nucleoporins and Transport Receptors in Cell Differentiation

**DOI:** 10.3389/fphys.2020.00239

**Published:** 2020-04-03

**Authors:** Asmat Ullah Khan, Rongmei Qu, Jun Ouyang, Jingxing Dai

**Affiliations:** Guangdong Provincial Key Laboratory of Medical Biomechanics, Department of Anatomy, School of Basic Medical Science, Southern Medical University, Guangzhou, China

**Keywords:** nuclear pore complexes (NPCs), differentiation, nucleoporin, nucleocytoplasmic transport, nuclear membrane

## Abstract

Bidirectional molecular movements between the nucleus and cytoplasm take place through nuclear pore complexes (NPCs) embedded in the nuclear membrane. These macromolecular structures are composed of several nucleoporins, which form seven different subcomplexes based on their biochemical affinity. These nucleoporins are integral components of the complex, not only allowing passive transport but also interacting with importin, exportin, and other molecules that are required for transport of protein in various cellular processes. Transport of different proteins is carried out either dependently or independently on transport receptors. As well as facilitating nucleocytoplasmic transport, nucleoporins also play an important role in cell differentiation, possibly by their direct gene interaction. This review will cover the general role of nucleoporins (whether its dependent or independent) and nucleocytoplasmic transport receptors in cell differentiation.

## Introduction

The nucleoplasm and cytoplasm are the main locations in which multiple vital processes within cells take place. These processes require bidirectional molecular trafficking between these domains, but free motion of molecules is restricted by the double membrane nuclear envelope. However, there are various nuclear pore complexes (NPCs) embedded in the nuclear pores present on the nuclear envelope. The NPCs are composed of several units. termed nucleoporins (Nup), which arrange structurally and form the main channels that facilitate transport between the nucleoplasm and cytoplasm (Kramer et al., 2008). Small molecules such as small proteins, metabolites, and ions can pass through these gates by passive diffusion, whereas large molecules require particular transport receptors, such as importins and exportins.

Nucleoporins in the NPC and their associated importins and exportins, also called karyopherin, are not only responsible for nucleocytoplasmic transport but are also involved directly or indirectly (dependently/independently on transport receptors) in various cellular processes, such as cell differentiation. Variations in the expression of nucleoporins were observed among different cell types (Guan et al., 2000) and tissues during the development (Jao et al., 2012; Zheng et al., 2012). Moreover, mutations in several nucleoporins are reported to be involved in several diseases (Cronshaw and Matunis, 2003; Neilson et al., 2009). In this review, we will specifically discuss the general role of NPCs and transport receptor proteins in cell differentiation.

## Nuclear Pore Complex

### Structure and Composition

**FIGURE 1:**
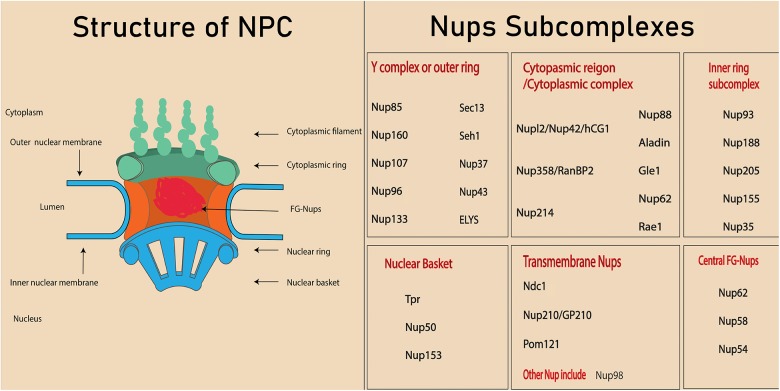
Structure of nuclear pore complex and Nups subcomplexes. Each nuclear pore complex (NPC) is embedded in grommet made by the outer and inner nuclear membrane. Phe-Gly (FG) Nups form the central channel of the nuclear pore complex. The peripheral structure of NPC consists of cytoplasmic filament, nuclear filament, and nuclear basket.

About 3000 NPCs can be found on the nuclear membrane of a mammalian cell (Dultz and Ellenberg, 2010). These complexes are termed the gatekeepers of the nucleus because of their involvement in almost all nucleocytoplasmic transport. The NPC is an eightfold symmetrical structure with a molecular weight of 60–125 MDa and consists of 30 different nuclear proteins, which are collectively called nucleoporins or Nups (Alber et al., 2007). It has been demonstrated in a cryo-electron tomographic study that NPCs constitute three distinct regions in the nuclear membrane: (1) the nuclear basket (so-called because of its basket-like shape), (2) the cytoplasmic ring from which filaments extend into the cytoplasm, and (3) the central framework which makes up the pore (Hurt and Beck, 2015). Nups form various subcomplexes (Figure 1; Kabachinski and Schwartz, 2015; Beck and Hurt, 2017) based on their biochemical affinity for each other (Siniossoglou et al., 2000; Lutzmann et al., 2002).

Depending upon their location, Nups will contain different types of structural domains such as alpha helices, β propellers, and phenylalanine-glycine (FG) repeats (Hoelz et al., 2011). About one third of the NPC is composed of special types of Nups called FG Nups, which fill the central channel of NPC. These Nups are assumed to have an unfolded structure that helps them bind with the transport receptors (Isgro and Schulten, 2007).

Different subcomplexes, mentioned in Figure 1, constitute the structure of NPCs by arranging in a particular way that means the cytoplasmic region is on the cytoplasmic side, while the nuclear basket is on the nuclear side. Membrane Nups help in anchoring the NPC into a nuclear envelop, while FG Nups directly lie in the inner most of NPC and directly interact with the transport. The structure NPC is highly conserved in eukaryotes and investigation conducted on yeast and eukaryotes indicated the similarity in the localization and function of NPC, even though the sequences are not exactly conserved. In fact, the nucleocytoplasmic transport through NPC and Nups facilitate various cellular processes (Jamali et al., 2011). Since various nucleoporin are also crucial in tissue and organs, their absence or mutation leads to the defect in the organ’s development (Zheng et al., 2012; Niu et al., 2014). The structure of an NPC and its Nups are involved in various cellular processes such as chromatin regulation, cell proliferation, cell differentiation, and cell fate. For example, a few high mobile nucleoporins change their localization from NPC to nucleoplasm and are involved in chromatin interaction (Liang et al., 2013). In addition, some nucleoporins are also involved in the direct association with cell signaling protein (Yang et al., 2015, 2019). Other components of nucleocytoplasmic transport, the transport receptors, play a crucial role in the transport of proteins, including transcription factors and signaling molecules (Yang et al., 2014). Interaction between transport receptors and nucleoporins is very important in transportation of these proteins in various cellular processes, including the cell differentiation. This review will cover the role of various Nups and transport receptors in cell differentiation.

## Nucleoporin Subcomplexes in Cell Differentiation

### Y-complex/Outer Ring Subcomplex

Recent studies have shown that, as well as their role in cytoplasmic transport, expressions of nucleoporins are changed during cell proliferation and differentiation, and thus may be involved in determining cell fate (Lupu et al., 2008; Gomez-Cavazos and Hetzer, 2015). A study of the role of Nups in the differentiation of mouse embryonic stem cells (ESCs) showed that mouse ESCs with depleted Nup133 were not only inefficiently differentiated into the neural lineage but also sustained abnormal pluripotency features (Oct4). A decline in the capacity of generation of post-mitotic neurons was observed in the Nup133 deficient neural progenitor cells, which pointed to its role in the development requirement for the establishment of neural lineage (Lupu et al., 2008). Study on Hela cells have not revealed any involvement of Nup133 in the transport of nuclear protein through NLS (nuclear localization signal) and NES (nuclear export signal) (Vasu et al., 2001; Walther et al., 2003). In addition, the localization of NLS dependent transcription factors Oct4, Nanog, and Bern2 (Pan et al., 2004; Do et al., 2007; Yasuhara et al., 2007) were also normal in Nup deficient cells. Moreover, similar expression and localization of importin α1, and importin α5 in merm (mermaid) and wild-type embryos was observed. Although Nup133 does not influence NLS dependent protein transport, it might be possible that it regulates a subset of proteins using NLS in the independent way (Lupu et al., 2008).

Another nucleoporin, Seh1, was found to be associated with a missing oocyte (mio) gene, which is required for the maintenance of meiotic cycle and oocyte fate during differentiation. The level of mio protein was reduced in the mutant Seh1 oocyte, thereby oocyte failed to differentiate and become psuedonurse cells (Senger et al., 2011). Nup107 in the same complex influenced the activity of somatic cells by regulating the expression of gonadal specific genes or by interacting with the tissue specific factors, that are imperative for the differentiation of nurse cells and oocyte (Weinberg-Shukron et al., 2015). Nup107 was also found to influence chondrogenic differentiation in the zebrafish embryo; an embryo deficient in Nup107 led to impaired chondrogenic differentiation during pharyngeal arch formation (Zheng et al., 2012).

Nup96 regulates T and B cell proliferation in innate and adaptive immune systems. Mice with lower expression levels of Nup96 showed compromised T cell proliferation because of a decrease in the expression of interferon-regulated genes (Faria et al., 2006). Sec13 interacts with Nup96; therefore, the same trend was observed in a study conducted on mice with low levels of Sec13 (Moreira et al., 2015). Sec13 may have a role in the differentiation of retinal cells, according to a study conducted on Sec13^sq198^ mutant zebrafish (Niu et al., 2014). Surprisingly, another member of this complex, ELYS, influenced the proliferation and development of neuronal, retinal, and intestinal cells of zebrafish (Davuluri et al., 2008; de Jong–Curtain et al., 2009).

### Cytoplasmic Region

Nup358/RanBP2 is associated with muscle cell differentiation, and the depletion of Nup358 inhibits the formation of myotubes. During differentiation, the structure of the NPC was observed to be changed, and Nup358 was the key component in this remodeling of the architecture of NPC (Asally et al., 2011). Differentiation of hippocampal axon-dendritic neurons is also regulated by Nup358, via interactions with Disheveled (a mediator of Wnt signaling) and aPKC (Par polarity complex) (Vyas et al., 2013). Gle1 was found to have a role in motor-axon arborization and, as in Gle1-deficient zebrafish, neural precursors failed to differentiate into the terminal stages (Jao et al., 2012). Schwann cells require Gle1 for differentiation and proliferation from their precursor cells into myelinating Schwann cells (Seytanoglu et al., 2016). Perez-Terzi (Perez-Terzic et al., 2003) studied the role of NPC structure and various Nups and transport receptors in stem cell-derived cardiomyocytes. Alteration of the structure of the NPC, nucleoporin expression (Nup214, Nup358, Nup153), and p62 (involved in nuclear transport) facilitate the differentiation of ESCs into the cardiac lineage (Perez-Terzic et al., 2007). Another important nucleoporin, Rae1, was found to be involved in germ line differentiation in testes of *Drosophila*, suggesting the importance of this protein in male fertility (Volpi et al., 2013).

### Inner Ring Subcomplex

In this complex, the expression of Nup155 was observed to be highest in the skeletal and cardiac muscle cells. Nup155-depleted mice were shown to die in early embryogenesis, indicating its significance in embryonic development. The heterozygous recessive gene of Nup155 was associated with an atrial fibrillation phenotype (Zhang et al., 2008). Expression of Nup93 was observed in all cell types of kidney cells, and its mutation leads to the pathogenesis of focal segmental glomerulosclerosis (Hashimoto et al., 2019). Knock down of Nup93 causes the reduction in human podocyte proliferation. Interaction of Nup93 and XPO5 was observed with SMAD 4 signaling protein. Mutation in Nup93 leads to the abrogation of SMAD activity which causes Steroid Resistant Nephrotic Syndrome (SRNS) (Braun et al., 2016). Nup35 regulates the cardiac pH by regulating the membrane protein NHE1expression (Xu et al., 2015).

### Nuclear Basket

Surprisingly, Nup50 and Nup153 are found in both the NPC and in the nucleoplasm, and they are thus considered mobile nucleoporins whose movement can be detected in various locations (Buchwalter et al., 2014; Kitazawa and Rijli, 2017). For example, Nup50 was shown to promote differentiation of myoblasts during the development of myotubes with its transport independent role (Buchwalter et al., 2014). Nup50 was found to be downregulated along with Kifc1 (kinesin super family) during muscle cell differentiation in C2C12 cells.

Nup50 relies on Nup153, as depletion of Nup153 causes displacement of Nup50 from the NPC (Hase and Cordes, 2003). In human ESCs, depletion of Nup153 induced early differentiation and was found to be involved in silencing the developmental gene (neural specific genes: Pax6, Blbp, Nes, and Tubb3) without altering nucleocytoplasmic transport (transport independent). It binds with the transcriptional start site (TSS) of developmental genes and facilitates the recruitment of polycomb repressor complex 1 (PRC1) (Jacinto et al., 2015). Nup153 interacts with transcriptional factor Sox2 and is downregulated during the differentiation of neuronal progenitor cells into neurons (Kitazawa and Rijli, 2017). Nup153 not only interacts with Sox2 in neuronal differentiation and cell proliferation (Smitherman et al., 2000; Toda et al., 2017), but also interacts with and co-regulates other genes, suggesting it might contribute to determining neural fate (Toda et al., 2017).

### Central FG-Nups and Transmembrane Nups

Among the central FG-Nups, Nup62 plays an important role in embryonic development (Convergence and extension of gastrula, dorsoventral patterning, and specification of midline organ precursor) by recruiting β-catenin in the Wnt signaling pathway, and also regulates Wnt/β-catenin and the BMP signaling pathway (Yang et al., 2015).

Nup210 is mobile, dynamic (Rabut et al., 2004), and highly expressed in many organs, including the brain, skin, lung, kidney, pancreas, and gut (Olsson et al., 1999). Nup210 was first studied because of its variation in expression during epithelial differentiation in metanephric epithelial differentiation. Nup210 was found to be essential not only for myogenic differentiation, but also for neuronal differentiation. It was found to be expressed not during the proliferation of ESCs and myoblasts but at the time of cell differentiation. The dynamic nature of Nup210 is indicated by the fact that depletion of Nup210 had no influence on nucleocytoplasmic transport but caused downregulation of several genes associated with differentiation (D’Angelo et al., 2012). Comprising a luminal domain and C-domain, Nup210 plays its part in differentiation through the luminal domain, which is continuous with the endoplasmic reticulum. It has been suggested that Nup210 regulates calcium homeostasis in the endoplasmic reticulum during muscle differentiation. However, further studies are required to determine the effects of Nup210 binding to calcium muscle differentiation (Gomez-Cavazos and Hetzer, 2015). NDC-1 interacts with SEPTIN to form a complex that is co-expressed at the manchette and neck region of sperm during terminal differentiation in mammalian spermiogenesis (Lai et al., 2016).

### Other Nups: Nup98

The effects of Nup98 and Nup96 on the proliferation and differentiation of the germ line were investigated in *Drosophila* (Parrott et al., 2011). The results revealed that the Nup98-96 genes are required for the cell to be in an undifferentiated state. Specifically, Nup98-96 are required for cell proliferation in the germ line, which in turn prevents the cell from differentiating. The binding of the mobile Nup98 to genes linked with development and differentiation in ESCs was studied by Liang et al. (2013). Nup98 was observed to bind to and activate neural developmental genes during the neural differentiation of ESCs.

### Role of Nups in Chromatin Regulation

Recent studies have suggested that mobile Nups (e.g., NUP50, NUP153, and NUP98) can directly associate with chromatin because they can shuttle from nucleoplasm to NPC (Griffis et al., 2002, 2004; Buchwalter et al., 2014)., Nup50 is a mobile Nup independent of its role in nuclear transport, and promotes muscle cell differentiation by its direct interaction with chromatin (Buchwalter et al., 2014). Another mobile Nup, Nup98, also interacts with the chromatin during the embryonic stem cell differentiation. Nup98 interacts with genes at the NPC in the early stage of development, whereas in the later stages interaction of highly inducible genes are reported to occur in the nucleus interior (Liang et al., 2013). In Hela cells, a nuclear basket nucleoporin Tpr is essential for heterochromatin organization, while deleting Tpr results in eliminating heterochromatin from free regions at NPC (Krull et al., 2010). Moreover, Nup153 regulates heterochromatin domain in interphase cells by recruiting Repo-man (CDCA2: Cell division cycle-associated protein), a protein phosphatase 1 (PP1)-targeting subunit protein, that ultimately leads to chromatin remodeling (De Castro et al., 2017; Pascual-Garcia et al., 2017; Rowley and Corces, 2018; Tan-Wong et al., 2009). Furthermore, NPC as a whole entity acts as a scaffold for the protein mediating epigenetic mechanism. These proteins include chromatin architectural protein, chromatin remodeling, and histone modifying proteins that influence the organization of chromatin (Bickmore and van Steensel, 2013). In mitogen activated cells, Nup153 and Tpr recruit an MYC transcription factor that is highly expressed in cell proliferation (Su et al., 2018). Transmembrane Nup210 is also involved in the regulation of several genes seemingly transporting independent manner (D’Angelo et al., 2012). These data provide potential interest for future studies on the molecular role of nucleoporins and their interaction with chromatin regulation. How this interaction impact various cellular processes may need a comprehensive study.

## Role of Npc in Protein Transport

The disorderly arrangement of FG-Nups leads to the formation of a permeability barrier in the NPC. Facilitation of the transport of specific proteins is controlled by the FG-Nups, allowing the cell to regulate the movement of molecules at the nucleoporin level. Nucleoporins contribute to the translocation of specific molecules carrying a peptide sequence that is recognized by the karyopherin (transport receptors) (Flores and Seger, 2013; Ma et al., 2016). Transport receptors have important roles in nucleocytoplasmic transport and can be classified into specific transporter protein types, namely importin and exportin (nuclear transport receptors). Based on their physiological composition, importins are further divided into two types: importin α and importin β. Importin α facilitates the movement of nuclear localization signals (NLS) (monopartite PKKKRKV and bipartite KRPAATKKAGQAKKKK) (Poon and Jans, 2005) through the NPC by forming a trimeric complex with the cargo molecule (NLS) and importin β1.

Thus, NLS-importin and importin β1 interact with multiple FG-Nups of the NPC during translocation into the nucleus. Strong interactions of importin β have been observed with various FG-Nups: Nup358, Nlp1, Nup214, Nup98, Pom121, Nup58, Nup54, Nup62, Nup153, and Nup50. Importin β also has strong interactions with Nlp1, Nup98, and Nup54, whereas weaker binding is observed with Nup358 and Nup58 (Ma et al., 2016). On the nuclear side of the NPC, RAN-GTP dissociates the cargo molecule by interacting with the trimeric complex. FG-Nups influence the disassociation of this cargo molecule from the importin α by interacting with the complex (Lindsay et al., 2002; Matsuura and Stewart, 2005). In the presence of RAN-GTP, importin α is exported back to the cytoplasm in complex with cellular apoptosis susceptibility protein (CAS), and importin β1 is recycled back with RAN-GTP (Stewart, 2007; Wente and Rout, 2010; Miyamoto et al., 2012).

**FIGURE 2:**
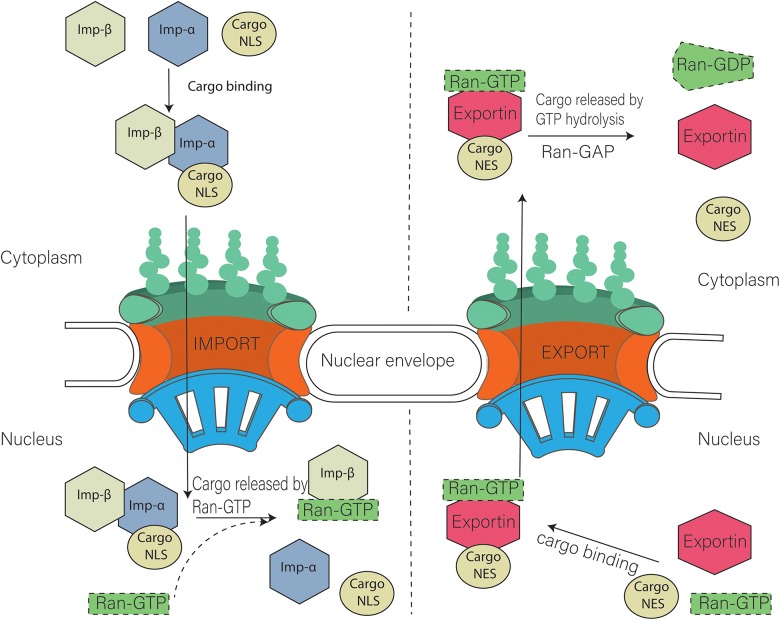
Model of nuclear import and export. Cargo containing NLS (Nuclear localization signal) is imported with the help of Importin α and importin β heterodimer. Nuclear export of cargo having NES (nuclear export signal) is carried out with the help of exportins. Ran GTP is also required during that process.

Nuclear export requires recognition of a peptide sequence on the Nuclear export signal (NES i.e., RFLSLEPL and TPTDVRDVDI in cyclin D (Poon and Jans, 2005) and LQKKLEELEL in mitogen-activated protein kinase) (Kutay and Güttinger, 2005) cargo by the exportin, which attaches to the RAN-GTP. This complex interacts with the Nup for translocation to the cytoplasm. GTPase is required to ensure the disassembly of this exportin-cargo complex in cytoplasm. Ran GTPase ensures the directionality of the transport and regulates transport receptors by promoting a gradient RanGTP–RanGDP across the nuclear envelope [(Low RanGTP) in the cytoplasm, (High RanGTP) in the nucleus]. The procedure is similar to nuclear import; the only difference is that the RAN-GTP binds to the complex at the start of export (Figure 2; Askjaer et al., 1998; Guan et al., 2000; Kutay and Güttinger, 2005).

Various models such as selective phase model, spaghetti oil model, virtual gate model, and reduction of dimensionality model have been proposed for use on the transport of molecules through central FG Nups. Most of the central Nups of NPCs contain many FG repeats; these Nups are aligned in such a way that they form a channel, with the FG repeats extending into the middle of the channel (approximately 200 FG-Nups per channel), thus establishing a permeability barrier in virtual gate model (Rout et al., 2003). Spaghetti oil model is based on the interaction of molecules with the nucleoporin for its translocation across the NPC. This model proposes the weak binding of nucleoporin and transport receptors during the molecular movement (Macara, 2001). In the selective phase model, central FG Nups make the gel like structure through their weak hydrophobic interaction. Small molecules (<5 nm) can pass through the sieve by passive transport, but molecules larger than 5 nm require active transport via interaction with the FG domain (Ribbeck and Görlich, 2001; Hülsmann et al., 2012; Labokha et al., 2013). The reduction of dimensionality proposes the movement of molecules by sliding on the surface of nucleoporin from the cytoplasmic face to the nuclear face (Peters, 2005).

## Role of Importins in Cell Differentiation

Importins can be further divided into three subtypes based on their amino acid sequences (Table 1). Subtype α1 consists of α5, α6, and α7, whereas α2 is comprised of importin α1 and importin α8, and the α3 subtype includes importin α3 and importin α4) (Pumroy and Cingolani, 2015; Miyamoto et al., 2016).

**TABLE 1:** Types of human importin alpha.

	**Importin α**
KPNA1	Importinα5
KPNA2	Importin α1
KPNA3	Importin α4
KPNA4	Importin α3
KPNA5	Importin α6
KPNA6	Importin α7
KPNA7	Importin α8

The importin α subtype expresses tissue specificity and influences the maintenance and formation of tissues. The structural dissimilarity among the importins alpha indicates that they regulate the movement of specific cargo proteins between the nucleus and cytoplasm (Köhler et al., 1997; Tsuji et al., 1997). Expression levels of different transporter proteins and their subtypes fluctuate during the differentiation of cells. For example, importin α1 expression at the mRNA level increased during the neural differentiation of neural stem cells (Ahn et al., 2004). Tissue-specific expression was observed during the neural differentiation of ESCs, wherein the importin α subtype switched to another subtype during neural development. Importin α1 was expressed more strongly in undifferentiated ESCs, whereas the level of importin α5 was higher in differentiated cells (Yasuhara et al., 2007). Kamikawa et al. (2011) studied the expression of KPNA2 (which encodes importin α 1) in ESCs and NIH3T3 cells and found KPNA2 to be higher in ESCs than in NIH3T3 cells. KPNA2 is activated by regulation of the transcription factors klf4 and klf2 (direct targets of Oct3/4), which helps maintain the ESC in the undifferentiated state.

Importins α1 and 2 (Da1, Da2) are also required for differentiation of stem cells of the germ line in *Drosophila*. The movement and expression of importin α is tightly regulated between nucleus and cytoplasm, because overexpression leads to death during the process of pupation (Gilboa and Lehmann, 2004; Ratan et al., 2008). The expression levels of importins α1, α2, and α3 changed with the passage of time during spermatogenesis. The expression of importin α1 and importin α was initiated in the starts of meiosis and ended during spermatid differentiation. Planaria (*Schmidtea mediterranea)* has two homologs of importin α, Smed-ima-2 and Smed-ima-1, which are important in stem cell survival and differentiation, respectively. It has been suggested that these homologs help to regulate importin α and nucleocytoplasmic transport, thus contributing to cell differentiation. The exact mechanism by which these homologs affect stem cell survival needs to be further understood (Hall et al., 2011; Hubert et al., 2015).

The importin α family also has an important role in the regulation of myogenesis, wherein myoblasts undergo differentiation, proliferation, and cell division. Myoblast proliferation has been found to be regulated both positively and negatively by KPNA1 (importin α5) and KPNA2 (importin α1), which suggests the import of particular cargo during proliferation. KPNA2 plays an important part in the regulation of myocyte migration, which is necessary for myogenesis. The roles of these importins in myogenesis are known, but how they adapt to their roles in aging and disease remain unknown. Importin α5 is essential for satellite cell proliferation and survival, as it helps to regulate the import of vital proteins and thus determine cell fate. Cells with depleted importin α5 exhibit premature activation and proliferation, leading to exhaustion and cell death. Moreover, critical protein cargoes are disrupted because of the impaired localization of importin α5 (Choo et al., 2016).

Importin α2 is the key importin that regulates the circadian clock in the cell by facilitating the localization of PER1/2 in the cytoplasm during stem cell differentiation (Umemura et al., 2014). The center of the circadian clock is in the supra-chiasmatic nucleus, although recent evidence indicates that the circadian clock resides in every part of the cell (Dibner et al., 2010). Various physiological processes, i.e., metabolism, differentiation, cell proliferation, and stem cell homeostasis, are controlled by the circadian clock. Comprehensive study is required to understand the role of nuclear cytoplasmic transport and the circadian clock in the differentiation of stem cells (Weger et al., 2017).

Regulation by importins of various pro-myelinating factors such as T3 and CNTF is essential for oligodendrocyte differentiation. Moreover, a change in Importin α response had been observed in factors responsible for differentiation. Upregulation of the expression of importin α3 has been observed with ciliary neurotrophic factor (CNTF) treatment, whereas inactivated importin α3 inhibits oligodendrocyte differentiation. Strong expression of importin α5 and 7 was observed with T3 treatment in oligodendrocyte differentiation (Laitman et al., 2017).

The expression of different importins not only varies among different tissues but also during the process of differentiation. Dexamethasone-induced differentiation of AR42J into acinar-like cells was associated with a stronger expression of importins α3 and α4 after induction, whereas importin α was not expressed at any stage. Induction of differentiation of HL60 cells to macrophage phenotypes and granulocytes was carried out using phorbol myristate acetate (PMA) and Dimethyl sulfoxide (DMSO), respectively. Importin α1 and 4 were strongly downregulated in both cases, whereas importins α3 and 5 were upregulated after PMA treatment (Koehler et al., 2002). Differentiation of HL60 into macrophages using 1,25 dihydroxyvitamin D3 (DVD) treatment resulted in downregulation of gene expression of importins α1, α3, β1, and β3, whereas importin α5 was upregulated. All-trans retinoic acid (ATRA)-induced differentiation into granulocytes resulted in the decreased expression of importins α1, β2, and β3. Importin α3 was downregulated during the initial period of differentiation, but values returned 72 h after induction. This up and downregulation of nucleocytoplasmic transport protein is an essential element in the differentiation of cells, but further studies are required for complete understanding of its role (Suzuki et al., 2008). Furthermore, the different expression levels of importins during the different stages of differentiation and their relationships with each other in stem cells need to be further elucidated.

## Importin β in Cell Differentiation

The importin β family includes not only the importins involved in the translocation of cargo into the nucleus, but also the exportins that are responsible for moving the cargo out of the nucleus (Table 2; Quan et al., 2008). Expression levels of different importin β family members are altered during the differentiation of ESCs. Moreover, the expression levels vary among different cell, e.g., IPO7, IPO11, XPO1, XPO4, and CSe1L have been shown to be more highly expressed in mouse/ESCs (mESc) compared with mouse embryo fibroblast cells. Different lineages from the differentiation of the same mESc cells are found to have different expression levels of importin β, e.g., neural ectoderm (NE) differentiated from the mESCs has the highest level of IPO13 importin β expression. Furthermore, meso-endoderm differentiated from mESCs has the highest expression of RanBP6, and the lowest expression of IPO11.

**TABLE 2:** Members of importin β family.

**Importin**	**Exportin**	**Bidirectional receptor/Shuttling receptor**
Importin-β/importin-β1 Transportin-1/importin-β2 Transportin-2/importin-β2b Importin-4 RanBP5/importin-β3/importin-5, importin-7, importin-8 Importin-9, importin-11 Transportin-SR/transportin- 3/importin-12	(CRM1/exportin-1, CAS/CSE1L/exportin-2, exportin-5, exportin-6, exportin-7, exportin-t, RanBP17	Importin-13 Exportin-4

Several genes of the importin β family are essential in maintaining the pluripotency of ESCs. Knocking down the three most highly expressed genes (importin 7, XPO4, and RanBP17) of the importin β family results in differentiation of cells. Importin β also contributes to lineage specification by regulating levels of transcription factors. Suppression of XPO4 and RanBP17 leads to the differentiation of mESCs into endoderm, while XPO4 and importin 7 are critical components for the initial and final stages of NE differentiation, respectively (Sangel et al., 2014).

Transportin plays a critical part in muscle cell differentiation by regulating the transport of the HuR protein between the nucleus and cytoplasm. Transportin 2 (importin β2b) upregulates HuR protein into the myoblast nucleus during the early stages of differentiation. However, cytoplasmic localization of the HuR protein is observed in differentiated cells (van der Giessen and Gallouzi, 2007). Transportin 2, along with Ranbp6 (Ran-binding protein), has been shown to be upregulated in cardiac cells derived from the differentiation of ESCs (Perez-Terzic et al., 2007). Another member of the importin β family, importin 13, is expressed in the brain, lungs, and eyes, and has an important role in differentiation and physiologic function. Limbal epithelial progenitor cells have high expression levels of importin 13, which plays an important role in the proliferation and differentiation of these progenitor cells (Wang et al., 2009; You et al., 2013). Cytoplasmic localization of importin 13 is essential during the initial stages of brain development, after which it is localized in the nucleus. This suggests its importance in brain development in mammals (Suzuki et al., 2006).

Expression of exportin during DVD-induced cell differentiation in HL60 cells was also studied, showing that exportin 1 and exportin t were downregulated during differentiation. Reduction in exportin 1 levels suppresses protein synthesis, which leads to growth arrest of cells (Ribbeck and Görlich, 2001). Reduction in expression of exportins, i.e., XPO1, XPO5, XPO6, XPO7, and XPOt, was also found both in DVD- and ARTA-induced HL60 differentiation (Suzuki et al., 2006). Exportin 1 and 7 have important roles in the process of erythroid differentiation. Exportin 1 regulates erythropoiesis by nuclear localization of HSP70. Exportin 7 also facilitates terminal differentiation via nuclear maturation in the erythroblast (Hattangadi et al., 2014).

How this nucleocytoplasmic transport affects the various cellular processes, specifically cell differentiation, can be explained by the fact that NPC is involved in the transport of different transcription factor/differentiation/pluripotency factors (Yang et al., 2014) which includes Oct4 (Lin et al., 2012), Sox2 (Baltus et al., 2009), Oct6, and Brn2 (Yasuhara et al., 2007). Different transport receptors were reported to be involved in the transport. For example, Oct4 which keeps the cells in an undifferentiated state and is shuttled by importin α, while Oct6 and Brn2 are necessary for the differentiation of ES cells into neural lineage. Importin α2/β1 shuttled Oct4 into the nucleus in the undifferentiated cells, whereas importin α4/β1 and/or importin α1/β1 shuttles Oct6 and Brn2 into the nucleus upon differentiation into neural lineage. Export of Oct4 from the nucleus to cytoplasm is carried out involving CRM export. A recent model suggests that Oct4 is exported by passive diffusion, however, the exact mechanism is not yet clear (Oka et al., 2013). Another transcription factor Sox2 is also imported by Importin β alone or Importin α5/β1 (Yasuhara et al., 2007). Importin α2 is essentially required for the maintenance of Oct4 expression in cells in the nucleus (Young et al., 2011). Export of Sox 2 was reported to be due to protein acetylation on the NES signal which mediates the nuclear export and degradation of sox2 in cytoplasm (Baltus et al., 2009). These studies suggest that transport receptor’s mediated transport of transcription factors might be the key for differentiation.

## Nucleoporins and Transport Receptors in Mesenchymal Stem Cell (Msc) Differentiation

Liu et al. (2010) conducted a study on the expression levels of various genes encoding the components involved in nucleocytoplasmic transport in the differentiation of MSC cells. Upregulation was observed for the genes that encode Nup37, Nup160, Nup98, Nup62CL, and Nup43, as well as importin α3, α4, α5, and α6, during osteogenic induction compared with the adipogenic condition. Adipogenic induction led to decreased expression levels of various nucleoporins (Nup50, Nup205, Nup188, Nup93, Nup153, Nup155, Nup88, Nup62, Nup214, Nup107, and Nup35) and transport receptors (KPNB1, IPO8, and IPO11) compared with the osteogenic condition. Importin 9 and exportin 6 are also important components in the differentiation of MSCs in osteogenesis. Importin 9 helps to translocate the actin G monomer from the cytoplasm to the nucleus, whereas exportin 6 is involved in the export of F-actin. Knocking down importin 9 led to the inhibition of osteogenesis, whereas the inhibition of exportin 6 stimulated the osteogenic process (Sen et al., 2017).

## Nucleoporin Associated Cell Signaling in Cell Differentiation

Nucleoporins are also involved in several cell signaling pathways that are essential for the survival, proliferation, and differentiation of cells. These include Wnt signaling, which relies on the β-catenin repeatedly interacting with nucleoporin (Sharma et al., 2016). Some studies have suggested that the movement of β-catenin occurs mostly via the transcription factor LEF 1 (lymphoid enhancer-binding factor 1) in a piggy-back manner (Huber et al., 1996; Kim and Hay, 2001). Recent studies on zebrafish reported that Nup62 was found to be involved in the activation of the Wnt pathway through the facilitation of the active import of β-catenin (Yang et al., 2015, 2019). Another important component of the Wnt signaling pathway, APC, which is critical for the maintenance and activation of β-catenin, was also found to be associated with FG-Nup153 and Nup358 (Collin et al., 2008). There might be a possibility of direct association between β-catenin and FG Nups that still needs further validation.

Modulation of the Wnt pathway is also involved in the differentiation of pluripotent stem cells, e.g., ESCs, into specific lineages. In intestinal stem cells, it helps to maintain homeostasis, whereas in hemopoietic stem cells, Wnt activation enhances cell proliferation (Bhavanasi and Klein, 2016). In mesenchymal stem cells, the Wnt signaling pathway was shown to be pro-osteogenic and anti-adipogenic (James, 2013). Further studies are required to understand the role of nucleoporins in mediating the Wnt signaling pathway and determining cell fate.

Another important pathway is extracellular signal-regulated kinase 1 and 2 (ERK1/2), which interacts with nucleoporin and influences nucleocytoplasmic transport. Nup214, Nup153, Nup30, and TPr are phosphorylated by interaction with ERK1/2. Nup is known to be a bona fide substrate of the ERK1/2 pathway. Interaction of ERK1/2 with Nup50 causes alterations in nucleocytoplasmic transport that results in impaired translocation of importin β. However, the molecular mechanism of this alteration in nucleocytoplasmic transport remains to be elucidated (Michailovici et al., 2014).

The ERK1/2 signaling pathway has an important role in cell proliferation and differentiation. In muscle progenitor cells, ERK is the key regulator between proliferation and differentiation. Translocation of ERK to the nucleus or cytoplasm leads to cell proliferation and differentiation, respectively (Michailovici et al., 2014). ERK signaling also has a mediating role in the differentiation of Bone Marrow Stem cell into endothelial cells induced by VEGF (Xu et al., 2008). ERK is a key regulator of switching between osteogenic and adipogenic differentiation in human MSC cells (Jaiswal et al., 2000). Further studies are required to investigate the roles of nucleoporins and signaling pathways in determining cell lineage.

## Conclusion and Future Perspective

Nucleoporins are not only involved in structuring the nuclear pore complex but also play an important role in translocating various molecules. Translocation of molecules requires the interaction of nucleoporins with importins and exportins. Several studies mentioned above have described the role of nucleoporins in cell differentiation, wherein they are directly required for many cells to differentiate into other cell types. Among the nucleoporins, several are confined to the NPC, however, a few mobile nucleoporins have been found in both NPCs and the nucleoplasm. This suggests that nucleoporins are involved not only in bidirectional transport but also in chromatin regulation. For example, Nucleoporin from the nuclear basket, being on periphery, have direct chromatin interaction which might modulate cell differentiation. Interestingly, the role of Nup153 and Nup50 in nuclear basket is independent of transport receptors, however, high-resolution imaging techniques is crucial to see the NPC-chromatin interaction. In addition, it is essential to understand the molecular mechanisms in genome regulation mediated by NPC and Nups and how these genome regulations subsequently influence the cell fate. Recently a comprehensive review (Donnaloja et al., 2019) regarding the prospective role of Nups in the mechano-transduction has been published. They proposed a model, wherein application of mechanics from the Sun1 protein of Linker of Nucleoskeleton and Cytoskeleton to Nup153 causes the nuclear stretch which ultimately affects the nuclear transport in mechanics. NPC could be the aspect in the field of mechanobiology which still lacks complete understanding.

Moreover, there are some Nups, e.g., Nup210 (transport independent) and Nup133, which are found to be positively regulated in the differentiated state of cells, which also indicates that different Nups might regulate differently to affect the cell fate. Furthermore, nucleoporin interactions with importin and exportin are required for transport of different protein/transcription factors essential for cellular processes. In addition, a few signaling molecules (e.g., β-catenin) have been shown recently to be regulated directly with FG nucleoporins (nup62) during nucleocytoplasmic transport. The role of FG-Nups and its interaction with receptors in the translocation of different protein molecules creates potential questions regarding how FG Nups interact with each other in this transport and how FG Nups show affinity to these particular transport receptors to regulate the transport from NPC in different cell processes. In fact, various studies in this review on nucleoporins and the transport concluded its importance in determining cell lineage during differentiation. However, further studies are required at a molecular level to investigate the role of nucleoporins, along with those of importin and exportin, in cell differentiation.

Limited data are available regarding the role of nucleoporin and nucleocytoplasmic transport in stem cell differentiation. For example, in mesenchymal cell differentiation, only a few studies have been carried out to determine the expression of Nups during the differentiation. Further investigations should be carried out to fully understand their role in the stem cell lineage. Furthermore, extensive studies are required on different transport proteins and their cargos to understand their roles in various cellular processes, including cell differentiation.

## Author Contributions

AK and JD generated the figures. JO and JD reviewed and suggested modifications to the content. JD designed the structure of review manuscript. All authors participated in the conception and writing of the manuscript.

## Conflict of Interest

The authors declare that the research was conducted in the absence of any commercial or financial relationships that could be construed as a potential conflict of interest.
